# Pulmonary Embolism and Chronic Superior Vena Cava Occlusion Complicating Central Line-Associated Venous Thromboembolism in a Sickle Cell Disease Patient

**DOI:** 10.7759/cureus.22113

**Published:** 2022-02-11

**Authors:** Ahmed Brgdar, Ademola S Ojo, Lamiaa Rougui, Kamrun Anee, Mahbubur Sumon, Alem Mehari

**Affiliations:** 1 Internal Medicine, Howard University Hospital, Washington, DC, USA; 2 Pulmonary and Critical Care, Howard University Hospital, Washington, DC, USA; 3 Internal Medicine, Jalalabad Ragib-Rabeya Medical College & Hospital, Sylhet, BGD

**Keywords:** sob - shortness of breath, superior vena cava (svc) obstruction, central venous line, venous thromboembolism (vte), sickle cell disease: scd

## Abstract

Sickle cell disease (SCD), the most common genetic disorder globally, is often associated with an increased risk of venous thromboembolic events (VTE). Many of these patients have central lines placed for the purposes of repeated medication administration, blood transfusions, and blood draw, further increasing the risk of VTE. Given the non-specific presentation of VTE and pulmonary embolism, as well as the risk of mortality if interventions are delayed, a high index of suspicion is required for early diagnosis of the condition. We report the case of a 35-year-old woman with SCD and a port-a-cath in place who presented with extensive upper extremity and intrathoracic VTE with associated pulmonary embolism and chronic superior vena cava (SVC) occlusion. We also discuss the peculiarities of the clinical manifestations and management of VTE and pulmonary embolism in the setting of SCD based on the evidence from existing literature.

## Introduction

Sickle cell disease (SCD) is the most common genetic disorder globally with about 300,000 babies born annually with this condition [1]. According to the data from the CDC, about 100,000 Americans are affected by SCD with a prevalence of one in 365 among African-Americans and one in 16,300 among Hispanic-Americans [2]. The underlying genetic abnormality involves the replacement of glutamine with valine on position 6 of the beta chain [3]. Due to this abnormality, deoxygenated sickled hemoglobin polymerizes, forming long insoluble fibers, thereby leading to the deformation of red blood cells, sickling, and premature hemolysis, which underpins the myriads of clinical manifestations of this condition [3]. This multisystemic condition is mostly associated with painful and hemolytic crises; however, venous thromboembolism (VTE) is also a common manifestation [1].

There is an increased risk of venous thrombosis and pulmonary embolism in SCD patients with associated significant mortality [4]. A high index of suspicion is important for early diagnosis, and prompt intervention is warranted for optimal outcomes. In this report, we present a case of an SCD patient with extensive VTE involving the upper extremity and intrathoracic veins with chronic occlusion of the superior vena cava (SVC) and pulmonary embolism in the setting of an implanted vascular access device (IVAD, port-a-cath). We also outline the major peculiarities in the pathogenesis, clinical manifestations, and treatment of venous thrombosis and pulmonary embolism in SCD based on the evidence from existing literature.

## Case presentation

A 35-year-old woman presented to the emergency room complaining of shortness of breath and left-arm pain and swelling one day before the admission. She described her pain as throbbing, constant, and non-radiating. The pain was rated as 4/10 and without any relieving factors. She endorsed associated dyspnea, rhinorrhea, sore throat, and fatigue. Her past medical history was notable for SCD with hemoglobin SS and left intracranial ischemic stroke with complete recovery; she was on scheduled monthly exchange blood transfusions to prevent stroke recurrence. Vitals signs were significant for tachycardia and oxygen saturation of 94% on room air.

Physical examination showed a right chest wall port-a-cath, left upper extremity (LUE) swelling, tenderness noted in the LUE, palpable but weak radial and ulnar pulses in the LUE, and palpable dorsalis pedis pulses bilaterally. The skin was tight, and dusky/bluish tint color was observed in the LUE. The strength was normal in all extremities bilaterally.

**Figure 1:**
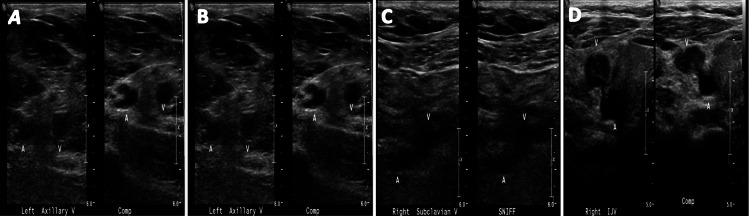
Doppler ultrasound of the left axillary vein (A), left brachial vein (B), right subclavian vein (C), and right internal jugular vein (D) illustrating non-compressibility consistent with thrombosis

The patient was admitted to the hospital. The severe acute respiratory syndrome coronavirus 2 (SARS-CoV-2) RNA test for coronavirus disease 2019 (COVID-19) was negative. Labs were remarkable for bicarbonate of 18 meq/L (normal range: 22-32 meq/L), LDH of 398 IU/L (normal range: 100-250 IU/L), total bilirubin of 3.4 mg/dl (normal range: 0.2-1.2 mg/dl) leukocytosis of 28.38 (normal range: 4-11 x 10^9^), hematocrit of 24%, reticulocyte count of 0.34 [normal range: (0.25-0.075) x 10], and reticulocyte percentage of 11.9% (normal range: 0.5-2%). A blood smear was remarkable for a few macrocytosis, microcytosis, few sickle and burr cells. Upper extremity Doppler showed extensive deep vein thrombosis (DVT) involving the left internal jugular, subclavian, axillary, brachial, and basilic veins in addition to thrombus in the right internal jugular and subclavian veins (Figure 1). Anticardiolipin antibodies, lupus anticoagulant, beta-2-glycoprotein I antibodies, factor V Leiden mutation, and prothrombin mutation screening were all negative.

**Figure 2:**
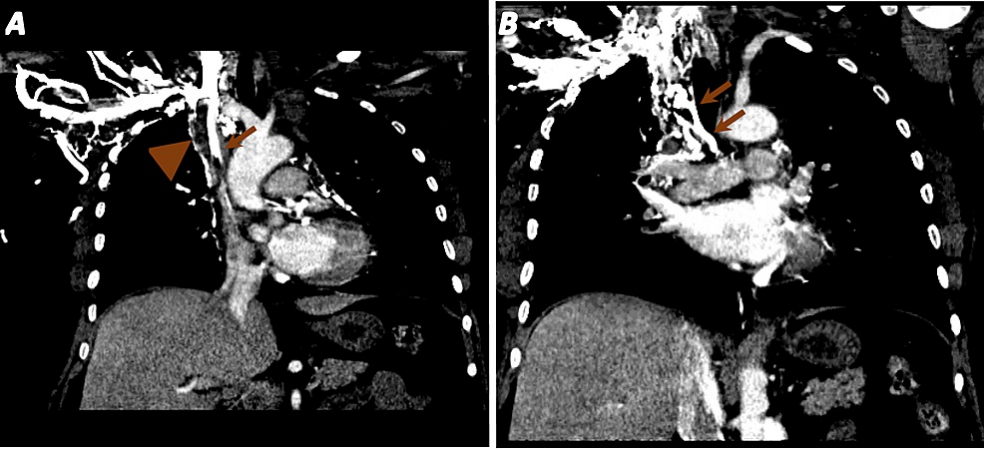
CT chest with contrast showing a filling defect in the superior vena cava (arrowhead) and a right-sided infusion catheter port with the tip at the cavoatrial junction (arrow) (A). Extensive chest wall and mediastinal collaterals (arrows) (B) CT: computed tomography

Contrast-enhanced CT scan of the chest showed pulmonary emboli in the right upper lobe and right middle lobe segmental pulmonary arteries. Thrombus of the SVC with extensive chest wall and mediastinal collaterals and thrombus in the azygos vein and left subclavian and axillary veins were also observed (Figure 2).

The patient was started on a heparin drip, which was later transitioned to subcutaneous low molecular-weight heparin. Her clinical condition improved on anticoagulation. However, due to chronic SVC occlusion and a high thrombus burden on the central line, the patient was scheduled for a bilateral upper extremity catheter-directed thrombolysis and subsequent percutaneous balloon venoplasty of the SVC and right and left subclavian veins. The patient tolerated the procedure well and heparin was transitioned to direct oral anticoagulants (DOACs) upon discharge. However, the port-a-cath was left in place as it was decided that removal and replacement would likely lead to similar outcomes. 

## Discussion

Virchow’s triad of hypercoagulability, endothelial dysfunction, and stasis, the underlying pathophysiology of venous thrombosis, is usually present in SCD patients, leading to an increased risk of thromboembolic events [5]. The average age at which venous thrombosis is diagnosed in the general population is 65 years, while the average age for high-risk thrombophilia is 30 years, which is comparable to the average age at which venous thrombosis of SCD is diagnosed [4]. A cross-sectional study of 404 patients at the Johns Hopkins Sickle Cell Center reported a median age of venous thrombosis diagnosis in SCD of 29.9 years, with 25% of the study population reporting a history of venous thrombosis [6]. Similarly, the prevalence of pulmonary embolism and mortality from the condition is higher in SCD than in the general population [7]. About 45% of individuals with pulmonary embolism have concomitant DVT [8]. Compared to the general population, there is an increased prevalence of pulmonary embolism in the absence of detectable DVT in SCD with in situ pulmonary thrombus formation or classic clot migration as the possible underlying etiology [4]. Our patient had extensive intrathoracic and upper extremity venous thrombosis. Not only is the risk of venous thromboembolic events higher in SCD, but the risk of arterial thrombosis is also equally increased, with stroke and silent cerebral ischemia as common manifestations in both children and adults with SCD [9,10]. Our index patient had experienced a prior ischemic stroke.

Most cases of pulmonary embolism in the setting of DVT occur following proximal infra-thoracic venous thrombosis (iliac, femoral, popliteal veins) [8]. DVT involving the intrathoracic veins (internal jugular, subclavian, brachiocephalic, azygos) are less common compared to venous thrombosis involving the lower extremity veins or internal iliac veins [11]. This is due to the effect of gravity on the venous circulation in the lower parts of the body. Most cases of intrathoracic venous thrombosis occur as a complication of vascular access procedures, such as dialysis catheter placement or central venous access [12]. Other less common associations are trauma, surgery, deep cervical infections, intravenous drug abuse, and malignancies [13,14]. There is a significant risk of pulmonary embolism in these individuals with central vein thrombosis [11]. However, the risk of pulmonary embolism is lower in upper extremity DVT with a reported incidence of only 2% [15]. Our patient had venous thrombosis involving both the upper extremity and intrathoracic veins in the setting of an IVAD (port-a-cath).

IVADs have significantly improved the management of patients who require long-term medication administration and repeated blood draws, such as cancer patients on chemotherapy and SCD patients who require long-term pain management [16]. However, the presence of these central lines constitutes a risk factor for upper extremity venous thrombosis with an incidence of 4-10% among cancer patients, although a lower risk has been reported among individuals with devices that have anti-thrombogenic properties [17,18]. The four common locations of central line-associated thrombosis are intraluminal, around the fibrin sheath formed at the tip of the catheter, central veins, and right atrium [19]. The presence of SCD and IVAD, therefore, increased the risk of intrathoracic and upper extremity venous thrombosis in our patient with a concomitant pulmonary embolism.

Based on the location of a thrombus within the pulmonary arterial circulation, pulmonary embolism could be classified as central or peripheral. Central pulmonary embolism involves the main pulmonary trunk and/or its two main divisions (right and left pulmonary arteries), while thrombi in the segmental or subsegmental pulmonary arteries are classified as peripheral pulmonary embolism [20]. Our patient presented with pulmonary embolism involving the subsegmental arteries of the right upper lobe pulmonary artery. These peripherally located emboli are often associated with more clinical signs of overt VTE than central pulmonary embolism and are more common in SCD [21]. Factors such as microvascular and small vessel disease in SCD in addition to hypercoagulability may be responsible for this peripheral predilection in SCD [22].

The non-specific presentation of acute pulmonary embolism means that many cases of pulmonary embolism are unrecognized or diagnosed late with serious sequelae. Sudden death is often the initial presentation in 30-50% of cases [23]. Dyspnea, tachypnea, tachycardia, and chest pain are the most common presenting symptoms [24]. The clinical picture is highly variable with manifestations such as respiratory failure, acute right heart failure, left heart failure, hypotension, syncope, atrial fibrillation, heart block, asthmatic like-syndrome, acute respiratory distress syndrome, positional dyspnea and hypoxemia, acute coronary syndrome-like chest pain, and delirium [25]. Pulmonary embolism is often initially misdiagnosed as acute chest syndrome in up to 25% of cases with SCD [26]. Therefore, a high index of suspicion is required for the diagnosis. Based on the history and physical examination findings, an attempt at determining the clinical probability of pulmonary embolism should be made [27]. The two most utilized scoring systems: the Wells score and the modified Geneva scoring system, are useful tools in establishing the clinical probability of pulmonary embolism, which can inform clinical decisions on further workup [28,29]. The combination of the information from these scoring systems and D-dimer levels increases the pretest probability of pulmonary embolism [27]. Other supportive laboratory evidence includes elevated troponin I, hypoxemia on arterial blood gas analysis, elevated brain natriuretic peptide (BNP) or proBNP, and echocardiographic evidence of right heart strain, although none of these is specific for pulmonary embolism [30]. Electrocardiographic abnormalities such as sinus tachycardia, new bundle branch block, right axis deviation, atrial fibrillation/flutter, premature atrial contractions, T-wave depression in leads V1-V4 and ST-segment elevation in V1 and aVR are often found in acute pulmonary embolism, but these are neither sensitive nor specific [31]. CT angiography (CTA) of the pulmonary vessels is the imaging modality of choice for the diagnosis of acute pulmonary embolism with high sensitivity and specificity [21]. The ventilation-perfusion (VQ) is an alternative to CTA although a normal scan does not entirely rule out CTA as up to 46% of cases with abnormalities of both ventilation and perfusion in the same area could show a non-diagnostic scan [32]. Magnetic resonance pulmonary angiography has a high sensitivity for detecting acute pulmonary embolism, although it is not often considered for that purpose due to the superiority and speed of obtaining a CTA of the pulmonary arteries [27].

Treatment of acute pulmonary embolism is determined by the hemodynamic status and the overall risk status of the patient [33]. For individuals with hemodynamic instability and the absence of contraindications, thrombolysis is the treatment of choice [34]. When it is contraindicated or not feasible, a surgical embolectomy or a percutaneous catheter-directed embolectomy is indicated [34,35]. Our patient had a chronic SVC occlusion and a high thrombus burden associated with the central line, necessitating catheter-directed thrombolysis and percutaneous balloon venoplasty for the SVC occlusion. Anticoagulation is the treatment of choice for both the acute and long-term management of pulmonary embolism regardless of the location of the thrombus. The conventional approach involves an initial heparinization during the acute phase and transitioning to warfarin for long-term management [36]. However, the rapid onset of action and effectiveness of the DOACs with good oral bioavailability, predictable pharmacokinetic profile, and reduced requirement for routine monitoring make them suitable for the acute and long-term management of pulmonary embolism [36]. Hence, they are the treatment of choice for the management of pulmonary embolism except in antiphospholipid syndrome, for which warfarin is preferred, and pregnancy, for which heparin is the treatment of choice [34]. The duration of anticoagulation depends on the risk of recurrence and is based on the inciting event (provoked versus unprovoked). At least three months of anticoagulation is recommended for individuals with a first-episode unprovoked pulmonary embolism, with a reevaluation for the need for anticoagulation after three months based on evidence from randomized controlled trials [37,38]. In the presence of IVADs such as the one reported in our index case, the current recommendation involves anticoagulation for at least three months [39]. Removal of the device is not recommended if the patient responds to anticoagulation and the device is functional, uninfected, and remains in an appropriate position [39]. All patients on anticoagulation should be closely monitored for any risk of bleeding [33].

## Conclusions

Venous thrombosis and pulmonary embolism are common disorders in patients with SCD. Their clinical manifestations are non-specific. Therefore, a high index of suspicion is required for prompt diagnosis and initiation of anticoagulation to increase the chance of optimal outcomes.
